# Equine dendritic cells generated with horse serum have enhanced functionality in comparison to dendritic cells generated with fetal bovine serum

**DOI:** 10.1186/s12917-016-0880-8

**Published:** 2016-11-15

**Authors:** Anja Ziegler, Helen Everett, Eman Hamza, Mattia Garbani, Vinzenz Gerber, Eliane Marti, Falko Steinbach

**Affiliations:** 1 Department of Clinical Research and Veterinary Public Health, Vetsuisse Faculty, University of Bern, Länggassstrasse 124, 3001 Bern, Switzerland; 2 Virology Department, Animal and Plant Health Agency-Weybridge, Woodham Lane, Addlestone, Surrey KT15 3NB UK; 3 Swiss Institute of Allergy and Asthma Research (SIAF), University of Zürich, Davos, Switzerland; 4 Swiss Institute of Equine Medicine, University of Bern, Bern, Switzerland; 5 Faculty of Health and Medical Sciences, School of Veterinary Medicine, University of Surrey, Daphne Jackson Road, Guildford, GU2 7AL UK; 6 Department of Zoonoses, Faculty of Veterinary Medicine, Cairo University, Cairo, Egypt

**Keywords:** Dendritic cell, Horse, Fetal bovine serum

## Abstract

**Background:**

Dendritic cells are professional antigen-presenting cells that play an essential role in the initiation and modulation of T cell responses. They have been studied widely for their potential clinical applications, but for clinical use to be successful, alternatives to xenogeneic substances like fetal bovine serum (FBS) in cell culture need to be found. Protocols for the generation of dendritic cells ex vivo from monocytes are well established for several species, including horses. Currently, the gold standard protocol for generating dendritic cells from monocytes across various species relies upon a combination of GM-CSF and IL-4 added to cell culture medium which is supplemented with FBS. The aim of this study was to substitute FBS with heterologous horse serum. For this purpose, equine monocyte-derived dendritic cells (eqMoDC) were generated in the presence of horse serum or FBS and analysed for the effect on morphology, phenotype and immunological properties. Changes in the expression of phenotypic markers (CD14, CD86, CD206) were assessed during dendritic cell maturation by flow cytometry. To obtain a more complete picture of the eqMoDC differentiation and assess possible differences between FBS- and horse serum-driven cultures, a transcriptomic microarray analysis was performed. Lastly, immature eqMoDC were primed with a primary antigen (ovalbumin) or a recall antigen (tetanus toxoid) and, after maturation, were co-cultured with freshly isolated autologous CD5^+^ T lymphocytes to assess their T cell stimulatory capacity.

**Results:**

The microarray analysis demonstrated that eqMoDC generated with horse serum were indistinguishable from those generated with FBS. However, eqMoDC incubated with horse serum-supplemented medium exhibited a more characteristic dendritic cell morphology during differentiation from monocytes. A significant increase in cell viability was also observed in eqMoDC cultured with horse serum. Furthermore, eqMoDC generated in the presence of horse serum were found to be superior in their functional T lymphocyte priming capacity and to elicit significantly less non-specific proliferation.

**Conclusions:**

EqMoDC generated with horse serum-supplemented medium showed improved morphological characteristics, higher cell viability and exhibited a more robust performance in the functional T cell assays. Therefore, horse serum was found to be superior to FBS for generating equine monocyte-derived dendritic cells.

## Background

Dendritic cells are antigen-presenting cells specialized in uptake and presentation of antigens to T cells [1]. They are the only antigen-presenting cells capable of inducing primary immune responses in naïve T cells and are thus pivotal for the development of T cell responses [2, 3]. The function of dendritic cells is reflected in a number of specific properties. Their distinct shape with many cellular processes offers a large surface area for antigen recognition and uptake [4]. Furthermore, the high surface expression of MHC class II in connection with high levels of costimulatory molecules allows for optimal stimulation of T cells. Initially, studies using dendritic cells have been hindered by difficulties in obtaining sufficient numbers of these cells, as their frequency is very low (<1%). The discovery that granulocyte-macrophage colony stimulating factor (GM-CSF) was the key cytokine needed to differentiate viable dendritic cells from murine blood [5] allowed the development of standardized methods to generate large numbers of dendritic cells ex vivo from hematopoietic progenitors. In humans, dendritic cells can be generated from peripheral blood CD14^+^ monocytes by using GM-CSF and Interleukin-4 (IL-4) [6, 7]. Due to the higher frequency of CD14^+^ cells, this method has been widely used to generate dendritic cells for experimental purposes in the human field and for immunotherapy. Monocyte-derived dendritic cells (MoDC) were shown to be homogeneous and could be fully matured using autologous monocyte-conditioned medium [8, 9] or, alternatively, through a cocktail of inflammatory cytokines, namely IL-1β, Tumor necrosis factor-α (TNF-α), IL-6 and Prostaglandin E2 (PGE_2_) [10]. The generation of MoDC has been described in a number of domestic animal species such as cattle [11], pigs [12], sheep [13] and horses [14–17].

Fetal bovine serum (FBS) represents an important source of nutrients for in vitro cell growth, metabolism and proliferation [18] and is widely used in cell culture media. Prior to the emergence of variant Creutzfeldt-Jakob disease as a result of the bovine spongiform encephalitis (BSE) crisis at the end of last century, FBS was considered reasonably safe for humans. In animals however, the safety of FBS was always more questionable with more animal diseases potentially being transmissible between species. The main advantage in using serum from unborn animals consists in the absence of interfering substances like inflammatory molecules, hormones or exogenous antigens, including feed-derived components. However, FBS batches are known to be heterogeneous in their performance and need to be batch-tested. Moreover, diluted and altered FBS has been sold recently in Europe, underlining the challenges to FBS selection [19]. Accordingly, FBS production is increasingly subject to regulations and restrictions, not least to protect animals under the 3R guidelines and reduce unnecessary pain, suffering, distress or lasting harm.

In recent years, the ex vivo generation of dendritic cells for the induction of anti-tumor responses has been a focus point for cancer immunotherapy research [20–23]. When generating dendritic cells for clinical applications, such as tumor vaccines, reproducibility and safety are of paramount importance. The use of FBS as a poorly defined cocktail of proteases and other active substances has always been less than ideal, and for both humans and animal species, the use of xenogeneic reagents needs to be avoided.

The utility of autologous serum or serum free media for the generation of MoDC has been questioned [24–26]. In horses, the use of homologous serum for cell culture has been described widely in systems other than DC generation or maintenance. Hamza et al. [27] used autologous serum in cell culture for functional assays involving equine peripheral blood mononuclear cells (PBMC). The use of horse serum for culture of primary equine bronchial fibroblasts [28] has also been described.

In the present study we examined the morphology, viability, phenotype and functional properties of eqMoDC generated under different serum conditions. For this purpose, three FBS batches from two different manufacturers were compared to horse serum produced in one of our laboratories. The data demonstrate that eqMoDC generated in the presence of heterologous horse serum perform equally well or better than dendritic cells generated with FBS.

## Methods

### Horses and blood samples

Blood samples were collected from the jugular vein of six healthy horses (4 geldings, 2 mares) using Sodium-Heparin containing vacutainers (Vacuette^®^; Greiner, St.Gallen, Switzerland). The horses were of diverse breeds (Warmblood, Freiberger, Icelandic horse) and spanned a large age range (4–25 years, mean age = 12.7 years). They had been vaccinated yearly against equine influenza and tetanus, and dewormed regularly. The horses belonged to the Swiss Institute of Equine Medicine, Vetsuisse Faculty, University of Bern.

### Horse and fetal bovine sera

For preparation of horse serum (HS), blood was collected from a healthy horse into Serum Clot Activator containing vacutainers (Vacuette^®^; Greiner, St.Gallen, Switzerland). HS was separated by leaving the blood to clot for 2 h followed by centrifugation at 2684×g (Rotanta 46 RSC centrifuge, Hettich AG, Bäch, Switzerland) for 10 min at 4 °C and inactivation for 30 min at 56 °C in a water bath. Serum was then stored at -20 °C until used.

Three commercially available batches of fetal bovine serum were used: FBS (A15-101), Lot A10106-1060 (PAA Pasching, Austria); FBS (S0113), Lot 1107A (Biochrom GmbH, Berlin Germany); FBS Superior (S0613), Lot 0503B, (Biochrom).

### In vitro generation of eqMoDC

PBMC were isolated by two sequential Ficoll density gradient centrifugations, using Biocoll 1.090 g/ml and 1.077 g/ml (Biochrom) as described by Mauel et al. [14]. The PBMC-containing fraction was then washed twice in PBS and re-suspended in PBS supplemented with 2 mM EDTA and 0.5% of FBS or HS. Monocytes were isolated by magnetic separation (MACS technology, Miltenyi Biotec GmbH, Bergisch-Gladbach, Germany) according to the manufacturer’s standard protocols, using a monoclonal anti-equine CD14 antibody (clone 105) [29]. Briefly, PBMC were first incubated with anti-CD14 and, after a washing step, with secondary goat anti-mouse micro beads. The cells were then separated on a LS column (Miltenyi Biotec GmbH). 2 × 10^6^ monocytes were incubated in a 12-well tissue culture plate (Falcon, BD Biosciences, San Jose CA, USA) in 2 ml RPMI 1640 medium with HEPES and L-glutamine (Gibco, Life Technologies Ltd, Paisley UK) supplemented with 1% penicillin and streptomycin (Gibco), 1% MEM vitamins, 1% Na pyruvate, 1% Non-essential amino acids (all Biochrom) and either 10% FBS (A15-101), FBS (S0113), FBS (S0613) or 5% HS.

Endotoxin contamination was assessed in all serum-supplemented media using a qualitative in vitro end-point endotoxin assay (ToxinSensor™, GenScript, Piscataway, NJ, USA). Lipopolysaccharide (LPS) levels were below 0.06 I.U./ml in all media. Differentiation into eqMoDC was induced by addition of 25 ng/ml recombinant (r.) equine GM-CSF and 10 ng/ml (r.) equine IL-4 (both Kingfisher Biotech Inc., St. Paul MN, USA) and cells were cultured for 3 days. Cells were monitored daily by light microscopy for changes in morphology.

### Viability assessment

Viability of three day old immature eqMoDC was assessed using the Alexa Fluor 488 annexin V/Dead Cell Apoptosis Kit for Flow Cytometry (Invitrogen Life Technologies, Paisley, UK) according to the protocol provided by the manufacturer. Briefly, cells were harvested from the cell culture plates, washed and an aliquot of cells was suspended in 0.4% Trypan Blue for counting using a hemocytometer (Neubauer). The cells were resuspended in annexin-binding buffer, incubated with 5 μl AF488 annexin V and 1 μl of 100 μg/ml propidium iodide for 15 minutes at room temperature, then analysed immediately by flow cytometry.

### Analysis of eqMoDC phenotype by flow cytometry

MoDC phenotypes were assessed by surface marker expression analysis using the following monoclonal antibodies: anti-equine CD14 mAb (clone 105) [29], anti-human CD206 clone 3.29B1.10 (Beckman Coulter, High Wycombe, UK) and anti-human CD86 clone IT2.2 (Becton Dickinson, Oxford, UK). Unlabelled antibodies were labelled using anti-mouse IgG1 zenon Kits (Invitrogen Life Technologies). Appropriate isotype controls were used. Analysis of cells was performed using a BD LSRII Flow Cytometer (BD Biosciences) and FlowJo software 6 (Tree Star Inc. Ashland OR, USA).

### Transcriptome analysis of eqMoDC populations

RNA was extracted from cell pellets of 1 × 10^6^ cells using the RNAqueous micro Kit (Life Technologies, Qiagen, Crawley, UK) and stored at -80 °C. RNA quality was assessed with the RNA 6000 Pico Labchip kit on the Agilent 2100 Bioanalyzer (Agilent Technologies, Berkshire, United Kingdom). The Ovation PicoSL WTA System v2 kit (NuGEN, Leek, The Netherlands) was used to amplify cDNA from 50 ng total RNA. The MinElute Reaction Cleanup Kit (Qiagen) option was used to purify cDNA, and 1 μg was then labelled using a one-color DNA labelling kit (NimbleGen, Madison, USA). For each sample, 4 μg labelled cDNA was hybridised to the NimbleGen 12 × 135 K custom equine arrays (Roche, Madison, USA). Three biological repeats were analysed for each data set. Hybridised arrays were scanned at 2 μm resolution with the Agilent High-resolution C Microarray Scanner (Agilent, Wokingham, UK). Microarray images were processed using DEVA v1.2.1 software (Roche, Madison, USA) to obtain a report containing the signal intensity values corresponding to each probe. The raw data was pre-processed using the DEVA v1.2.1 software by log2 transformation followed by RMA normalisation and summarisation to yield a signal intensity value for each probe set. The data set was then filtered by variance and Principal Component Analysis (PCA) was performed using Qlucore v2.0 software (Qlucore, Lund, Sweden). Variance levels were set using the δ/δ_max_ method.

### EqMoDC maturation and functional T cell stimulation assays

For antigen-presentation assays, immature eqMoDC were re-suspended at 2×10^5^ cells per 150ul of RPMI 1640 complete medium containing FBS or HS and incubated for 90 min at 37 °C either with 20 μg/ml tetanus toxoid (Schweizerisches Serum und Impfinstitut Bern, Switzerland) as a recall antigen, or 20 μg/ml of the primary antigen ovalbumin (OVA, kindly provided by the Swiss Institute for Allergy and Asthma Research, University of Zürich, Davos Switzerland), or with medium alone as a control. After antigen uptake, eqMoDC were washed and cultured in a 96-well round bottom tissue culture plate (Sarstedt, Nümbrecht, Germany) at 2×10^4^ eqMoDC per well in quadruplicates.

For maturation, antigen-primed eqMoDC were exposed overnight to a maturation cocktail comprising 1 μg/ml LPS (Sigma-Aldrich St. Louis MO, USA), 1 μg/ml PGE_2_ (Enzo Life Sciences, Exeter, UK), 20 ng/ml equine TNF-α, 10 ng/ml equine IL-1β, 20 ng/ml equine IL-6 and 100 ng/ml equine IFN-γ (all R&D Systems, Abingdon, UK) according to Moyo et al. [17].

Fresh CD5^+^ T lymphocytes were enriched by positive selection using MACS technology as above-mentioned, employing an anti-equine CD5 mAb (clone CVS5, Abd Serotec, Kidlington UK) and re-suspended in RPMI 1640 medium containing FBS or HS, respectively. Purity of CD5^+^ lymphocytes after bead selection was assessed by flow cytometry and was shown to be > 94%. 10^5^ autologous CD5^+^ T lymphocytes were added to the MoDC in the 96-well plate and co-cultured for 5 days at 37 °C/5% CO_2_. 5 μCi/well [^3^H] thymidine (Perkin Elmer, Waltham MA, USA) was added for the last 18 h of culture. DNA was then harvested onto a glass fibre filter plate and thymidine incorporation was measured on a scintillation counter (Inotech, LabLogic Systems Inc., Brandon FL, USA).

A mixed leukocyte reaction (MLR) was performed in addition to the autologous co-cultures by incubating matured eqMoDC with CD5^+^ T lymphocytes from another horse.

### Statistical analysis

Statistical analyses were carried out using the software program NCSS 8 (NCSS, Kaysville, Utah 84037, USA). Descriptive statistics showed that the data were not normally distributed. Therefore, non-parametric tests were used. The proportion of non-viable immature MoDC (Fig. 2), as well as the yield of immature eqMoDC (Table 1), was compared between the four serum culture conditions by using a non-parametric paired sample Wilcoxon (signed rank) test. The same test was used to determine differences in surface marker expression between eqMoDC before and after maturation (Fig. 3). A one-way ANOVA with Tukey-Kramer test for multiple comparisons was used to assess significant differences between the individual serum conditions within one maturation state (Fig. 3).

**Table 1:** Cell counts of immature eqMoDC generated after 3 days in vitro

Horse	HS	FBS (A15-101)	FBS (S0113)	FBS (S0613)
A	100	96	106.7	106.7
B	100	91.3	95.7	91.3
C	100	97.9	111.5	119.8
D	100	83.1	57.7	90
E	100	74.7	100.6	64.4
F	100	65.6	71.1	70.3
Mean (SD)	100 * (0)	84.8 * (12.75)	90.6 (21.37)	84.4 (21.02)

An identical number of monocytes from 6 horses (A-F) were differentiated in parallel and the resulting numbers of cells after three days counted. Results are shown as percentages with the number of cells generated with HS set as 100%. Using non-parametric paired Wilcoxon signed-rank test, only the comparison of HS vs. A15-101 was statistically significant (p ≤ 0.05), as indicated by the asterisks

Non-parametric paired sample Wilcoxon (signed rank) test was again used to assess differences in serum conditions with regard to induction of T cell proliferation by antigen-primed MoDC (Figs. 5 and 6). Overall, p-values ≤ 0.05 were considered significant.

## Results

### Morphology of differentiating eqMoDC differs between cells generated in the presence of HS or FBS

**Fig. 1:**
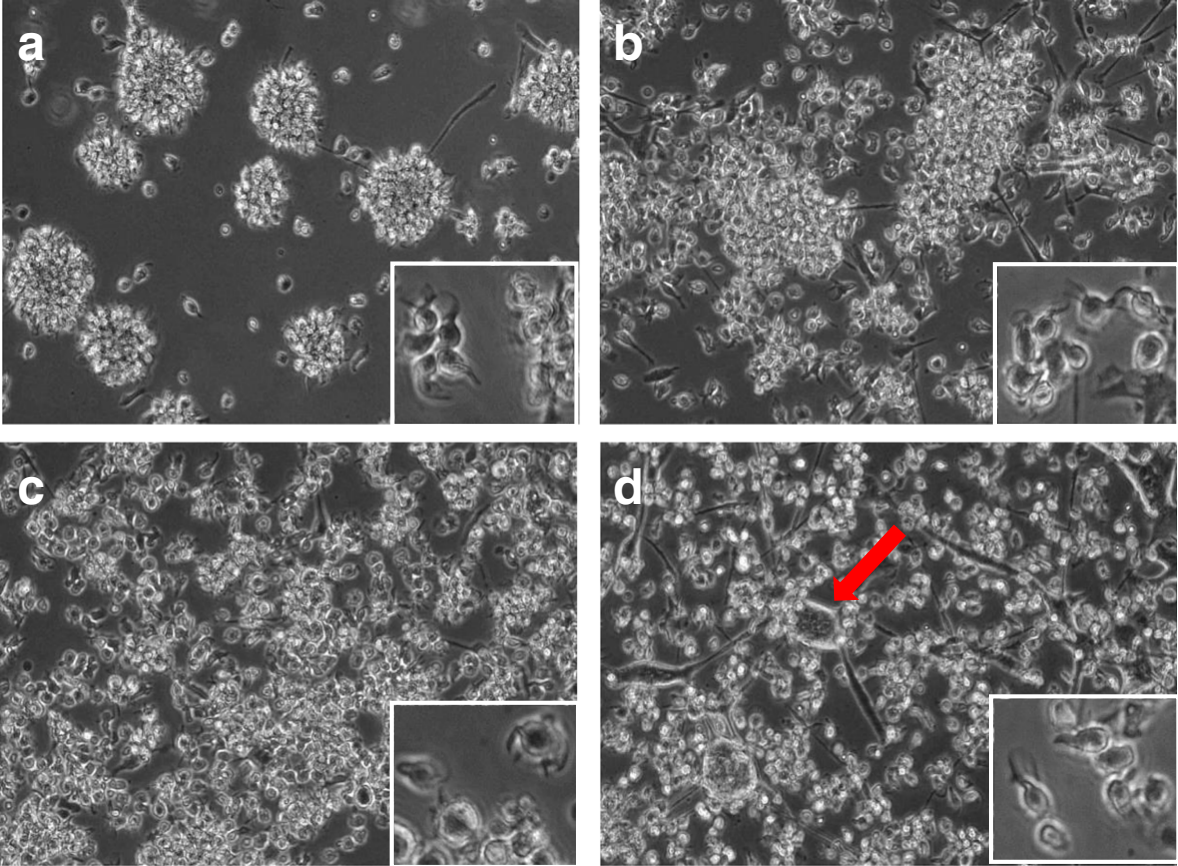
Morphology of eqMoDC at day 2 at 10× (large pictures) and 40× magnification (small inlaid pictures). Cells were incubated in the presence of HS (**a**), FBS batch S0113 (**b**), FBS batch S0613 (**c**) and FBS batch A15-101 (**d**), respectively. Pictures are representative of cells from 6 tested horses. The arrow in (**d**) indicates giant cells, presumably after uptake of apoptotic material, only seen in the FBS A15-101 condition

During differentiation from monocytes, MoDC undergo a distinct change in morphology. Equine CD14^+^ monocytes presented themselves as round cells that adhere quickly to cell culture plates. After 24 hours of stimulation, a formation of tight cell clusters could be seen. These became larger and denser as differentiation progressed (Fig. 1). Also, cellular dendrites could be seen by that time protruding from the clusters. After another 24 to 48 hours, the differentiated eqMoDC detached from the clusters and could be identified as large, heterogeneously formed cells with distinctive long dendrites. Marked differences in the morphology of the differentiating eqMoDC between serum conditions were observed. Cluster formation seemed to be impaired in all cells cultured with FBS in comparison to cells cultured in the presence of HS, where the vast majority of cells had formed into very distinct dense clusters (Fig. 1a). Cluster formation was less clear with both FBS from Biochrom (Fig. 1b, c) and almost non-observable with FBS batch A15-101 (Fig. 1d). Also, in the clusters that were present in FBS treated cells, fewer dendrites could be seen in comparison to HS. With FBS S0113 in particular, several long spindly cells were seen that could be an indicator of excess IL-4 in the culture [7]. However, identical concentrations of IL-4 were used for all serum conditions. When looking at individual cells in the 40× magnification (Fig. 1, small inlaid pictures), no significant differences could be determined between serum conditions. An intriguing finding with FBS A15-101 were large round objects enclosed by a clearly visible membrane, as indicated by the arrow in Fig. 1d.

### Immature eqMoDC generated in the presence of HS exhibit a higher viability

**Fig. 2:**
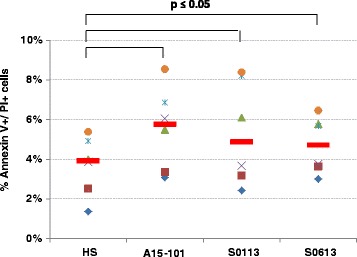
Proportion of non-viable immature eqMoDC measured by flow cytometry. The AF488 annexin V/Dead Cell Apoptosis Kit (Invitrogen) was used for the analysis according to the protocol provided in the kit. Each symbol indicates a separate horse (n = 6), with red lines indicating the median value. A non-parametric paired sample Wilcoxon (signed rank) test was used to determine significant differences between HS and the respective FBS condition. P-values ≤ 0.05 were considered statistically significant

It is essential for dendritic cells generated in vitro to maintain viability and thus be able to perform their functional tasks in downstream assays without becoming saturated with apoptotic material. Table 1 exhibits relative cell counts of differentiated eqMoDC following 3 days of incubation, starting with an identical number of cells for all serum conditions. Cell yield was significantly lower with A15-101 serum in comparison to HS. To assess differences in viability between the individual serum conditions, immature eqMoDC were tested using annexin V and propidium iodide staining and analysed by flow cytometry for the presence of apoptotic and/or dead cells. Figure 2 shows that cells cultured in the presence of any of the three FBS exhibited a significantly higher percentage of dead cells than cells cultured with HS. While differences between the individual FBS were non-significant, the highest proportion of dead cells could be observed in the cells incubated with the A15-101 serum.

### Surface marker expression of eqMoDC reflects maturation status and is comparable between serum conditions

**Fig. 3:**
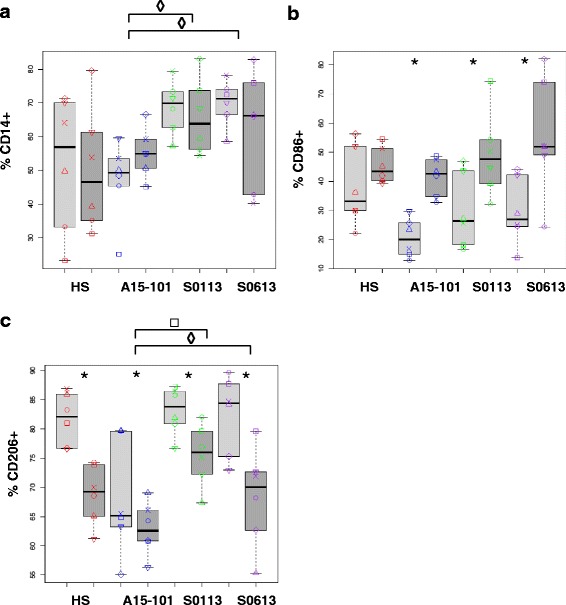
Surface marker expression of eqMoDC generated under the different serum conditions. EqMoDC were analysed by flow cytometry for cell surface expression of CD14 (**a**), CD86 (**b**) or CD206 (**c**). Results are displayed as percentage of positive cells for the respective marker. EqMoDC were generated under the respective serum conditions and were incubated overnight with the maturation cocktail (mature eqMoDC; dark grey boxplots) or maintained as immature eqMoDC (light grey boxplots). A non-parametric paired sample Wilcoxon (signed rank) test was used to identify significant differences between the HS condition and the different FBS conditions, respectively, within the maturation state (◊ indicates differences between immature eqMoDC; □ indicates differences between mature eqMoDC). The same test was also applied to investigate significant differences between immature and mature eqMoDC within each serum condition (indicated by asterisks). P-values ≤ 0.05 were considered statistically significant

Differentiation and maturation of eqMoDC in vitro is reflected by changes in the expression of surface markers. To compare our findings with previous results and established knowledge [17] CD14, CD86, and CD206 were selected for analysis by flow cytometry on eqMoDC before and after maturation and for comparison of eqMoDC generated under the different serum conditions. While CD14 is a monocyte marker that should be markedly down-regulated during the differentiation process from monocytes to MoDC, CD206 is a particular marker for immature MoDC to be down-regulated upon activation. CD86 was expected to be up-regulated during maturation/activation of MoDC. As expected, no significant difference could be observed in CD14 expression between immature and mature eqMoDC under any serum condition (Fig. 3a). Interestingly, CD14 remained significantly more highly expressed in immature eqMoDC obtained under the influence of the FBS batches (S0113) or (S0613) than in the presence of the A15-101 FBS batch. For CD86 expression, no significant difference between serum conditions could be observed (Fig. 3b). Nonetheless, eqMoDC incubated with the maturation cocktail showed an up-regulation of CD86 under all serum conditions. This up-regulation was statistically significant for FBS batches A15-101, S0113 and S0613. The difference in median values between immature versus mature eqMoDC was particularly low for HS. This was due to a considerable expression of CD86 already on immature eqMoDC. CD206 was clearly expressed upon differentiation of immature eqMoDC under all culture conditions, albeit at significantly lower levels in the presence of FBS A15-101 (median, range. 65.2%, 55.1–79.6%) compared to FBS batch S0113 (83.8%, 76.7–87.2%), or FBS batch S0613 (84.5%, 73–89.6) and also to HS (82.1%, 76.6–86.9%), although this difference was not statistically significant. A significant down-regulation of CD206 upon maturation could be observed in all four serum conditions (Fig. 3c).

### Transcriptome comparison of eqMoDC preparations and populations

**Fig. 4:**
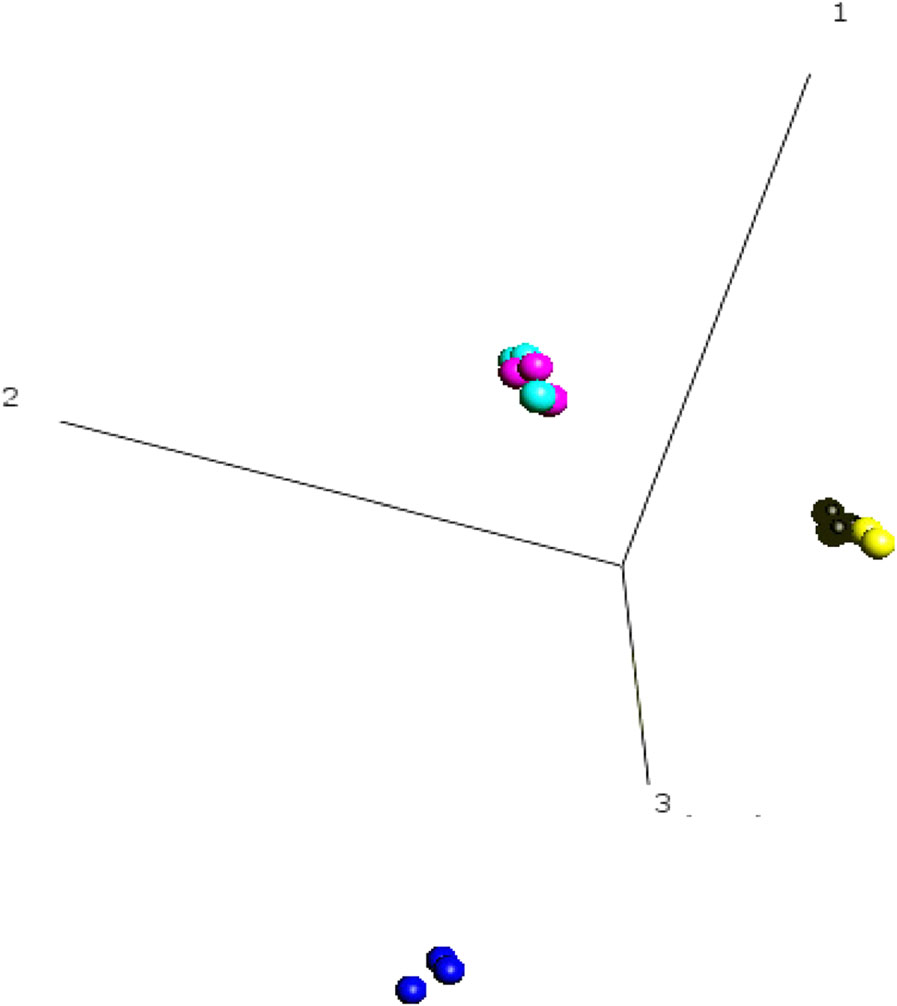
Gene expression analysis of equine monocytes (Mo) and eqMoDC. Mo (dark blue), iMoDC (black, FBS batch S0113; yellow, HS) and mMoDC (light blue, FBS batch S0113; pink, HS) were generated in parallel from three horses each. Principal component analysis (PCA) was carried out on the differentially expressed genes (1331 variables) and demonstrated that while the gene expression profiles of the different MoDC stages are separate and different to Mo, the genetic profile of the different sera cannot be further separated. Three biological repeats (horses) were analyzed using Qlucore, one dataset (iMoDC, HS) was discarded due to a technical issue in the hybridization of the sub-arra

While the above results demonstrate that HS provided results similar to FBS for the differentiation of equine eqMoDC, there is a considerable lack of antibodies in the horse system to perform a more comprehensive analysis. Aiming to test for hidden features, we resorted to gene expression profiling using equine-specific microarray analysis. Expression profiles of all three cell types were analysed by 3D PCA, which demonstrated that while monocytes (Mo), immature eqMoDC (iMoDC) and mature eqMoDC (mMoDC) are distinct populations, there was no detectable effect of the serum conditions on the gene expression profile of the different cell types (Fig. 4).

### Induction of T cell proliferation by antigen-primed MoDC

**Fig. 5:**
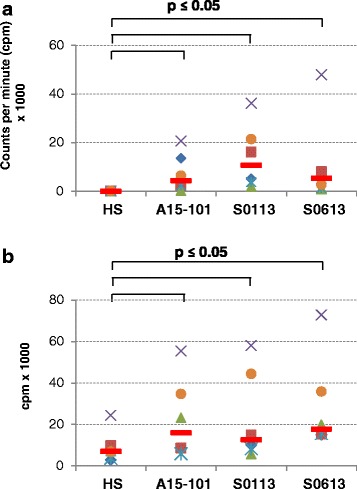
Non-specific background proliferation induced by the different serum conditions. T cell proliferation was measured by [^3^H] thymidine incorporation assay after a 5 day incubation of either CD5^+^ T cells alone (**a**) or co-incubation of CD5^+^ T cells with non antigen-primed eqMoDC (**b**). Each symbol indicates a separate horse (n = 6), with red lines indicating the median value. Non-parametric paired sample Wilcoxon (signed rank) tests were used to compare HS with each FBS and p-values ≤ 0.05 were considered statistically significant

**Fig. 6:**
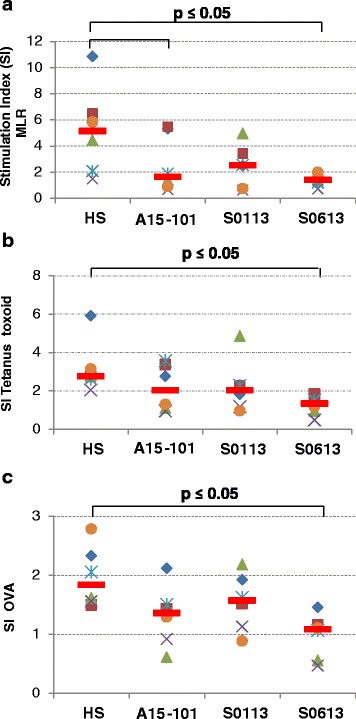
T cell proliferation induced by eqMoDC. T cells were stimulated with heterologous eqMoDC (mixed leukocyte reaction [MLR]) (**a**), as well as autologous eqMoDC primed with tetanus toxoid (**b**) or OVA (**c**). Results are displayed as stimulation indices (cpm with stimulation/cpm without stimulation). Each symbol indicates a separate horse (n = 6), with red lines indicating the median value. Non-parametric paired sample Wilcoxon (signed rank) tests were used to determine significant differences between the HS and the three FBS, respectively. P-values ≤ 0.05 were considered statistically significant

We analysed the eqMoDC generated under the different serum conditions for their ability to induce proliferation of T cells following antigen presentation. After the uptake of either tetanus toxoid as recall antigen, or OVA as a primary antigen, MoDC were incubated with autologous or heterologous (MLR) CD5^+^ T cells and proliferation of T cells was measured. While no proliferation could be observed in T cells incubated with medium only for the HS condition (median (range) 107 (67–170) cpm), T cells incubated with FBS showed a notable background proliferation (median (range) 4384 (300–20667) cpm with A15-101; 10709 (1935–36199) cpm with S0113; 5385 (1049–57954) cpm with S0613, respectively) (Fig. 5a). Similarly, non-specific proliferation of T cells co-incubated with non-primed MoDC was significantly higher in all FBS conditions (median (range) 15924 (5843–55404) cpm with A15-101; 12545 (5657–58043) cpm with S0113; 17719 (14952–72847) cpm with S0613, respectively) than in the HS condition (median (range) 6993 (2844–24414) cpm) (Fig. 5b). As expected, the strongest induction of T cell proliferation was observed with heterologous MoDC in the MLR control (Fig. 6a), followed by autologous tetanus toxoid-primed MoDC as a recall antigen presentation (Fig. 6b). T cell proliferation was least pronounced when using the primary antigen OVA (Fig. 6c). Serum culture conditions were compared for each antigen condition. The gradation between MLR, tetanus toxoid vs. OVA-elicited T cell responses was most evident in the HS condition, with horses exhibiting a median stimulation index for MLR of 5.14 (range 1.48–10.86) while for tetanus toxoid the SI was 2.75 (range 2.04–5.9). Three horses exhibited a stimulation index > 2 for OVA (median = 1.84; range 1.5–2.79). Use of HS in culture resulted in consistently higher stimulation indices when compared to the FBS conditions for MLR, tetanus and OVA, albeit not all differences were statistically significant.

## Discussion

Dendritic cells are key players in the immune system, particularly competent in modulating the immune responses [1]. Thus they are promising tools both for cancer immunotherapy and to limit immune responses to treat allergic reactions [30, 31]. The ability to generate these otherwise scarce immune cells in large quantities from progenitors and in particular monocytes, reinitiated research in this field around 20 years ago [6, 7, 32]. The identification of distinct DC populations in vivo, among them DC specialized in cross-presenting antigens to CD8^+^ cytotoxic T cells [33] has widened the opportunities to study specialized subsets of DC. MoDC remain the first choice for personalized therapeutic approaches, such as loading DC ex vivo, as they were shown to substitute for all DC functions, including cross-presentation [34].

Fetal bovine serum (FBS) is widely used in cell culture media, but has come under more intense scrutiny in recent years: as well as being a possible source for disease transmission its use in therapeutic vaccines may lead to adverse immune reactions against FBS [35, 36]. The problem of batch variability, including the contamination with LPS (which is detrimental to MoDC differentiation), was immediately recognised as an issue for therapeutic use of DC [37].

Early work to replace the 5–10% FBS commonly used to generate human MoDC by an equal amount of human sera (either autologous or batch tested) has not been successful (Steinbach et al., unpublished; various personal communications). This was explained by the plasticity of monocytes as uncommitted myeloid cells during differentiation allowing them to acquire a macrophage rather than a DC phenotype [38]. It was accordingly suggested to replace the 10% FBS by 1% autologous plasma, but while the phenotypical and functional data were analogous to FBS-derived MoDC, the DC yield obtained was very low (around 20% compared to FBS based protocols) with a significantly reduced enrichment [9]. This was offset by larger scale production [39], which, however, does not address the issue of cell debris from dead cells in such cultures.

In horses, previous studies to generate MoDC under the influence of GM-CSF and IL-4 used FBS [14, 16, 17] and pilot studies have also shown the potential use of such ex vivo generated MoDC for treatment of tumors [40]. However, previous experience where FBS-specific IgE was induced through MoDC application [36], eliminated such equine MoDC for the purpose of allergen immunotherapy.

In a preliminary experiment, we compared the use of autologous and heterologous equine serum with FBS from PAA (A15-101), which was used in one of our laboratories for the maintenance of cells lines. Intriguingly, while both equine serum preparation delivered encouraging results, the A15-101 FBS led to morphologically more heterogeneous populations with giant cells that we presumed to be the result of cell death followed by phagocytosis, not matching previous descriptions [14, 17]. Since the results obtained with the two serum preparations from horses were very similar, we decided to generate a heterologous serum to achieve a better standardisation for subsequent experiments. In addition, it became necessary to expand our study to other FBS batches able to reproduce previous results [17]. Thus, we decided to conduct a small study comparing three different FBS with HS during the generation of equine MoDC and their application in functional assays. The aim was to determine if equine MoDC could be successfully generated using horse serum that was freshly prepared rather than commercial serum that previously failed to deliver equine MoDC (Steinbach et al., unpublished).

The differentiation from monocytes to MoDC is characterised by the formation of tight cell clusters, where cells gradually become non-adherent and develop typical dendrites [6, 41]. It was notable that equine MoDC differentiated with HS developed larger clusters faster than monocytes incubated with FBS, which showed only limited clustering by day 2 (Fig. 1). It is not known whether the formation of cell clusters and thus close cell-to-cell contact is necessary for differentiating monocytes to become fully functional, but eqMoDC cultured in the presence of HS were also significantly more viable. It is thus likely that cell-cell signalling within clusters promotes viability of cells in culture. Not surprisingly and in line with the preliminary data, FBS batch A15-101 showed the highest proportion of non-viable cells. These results, however, do not explain whether HS contains additional viability factors which are lacking in FBS, or whether the changes and additions made to FBS batch A15-101 were detrimental to eqMoDC differentiation. Interestingly, there seems to be an inherent variation between individual horses in the proportion of non-viable cells that was independent of the serum used. Regardless of the serum condition, the same horse delivered the highest as well as the lowest proportion of dead cells. Thus, it is reasonable to propose that individual disposition (from genetics to an individual’s current health status) likely affects the generation of eqMoDC ex vivo. This resonates an earlier study showing that monocytes from Lupus Erythematosus patients required more GM-CSF and IL-4 to obtain viable DC [42].

As with the morphology and viability, clear differences were observed for the phenotype between the four tested sera: again, eqMoDC incubated with the two FBS from Biochrom displayed very similar phenotypes and maturation patterns, which were likely due to a similar FBS composition that should be observed using high performance FBS batches. Slightly surprising though was the relatively high level of CD14 remaining on MoDC generated with the Biochrom FBS. Since we tested all media for LPS this can be excluded as the causative factor. MoDC generated with HS expressed slightly higher levels of CD86 already at the immature stage. Overall, though all cells displayed a phenotype in accordance with MoDC differentiation and maturation and only trends were observed that made cells treated with HS preferable, i.e. is more in line with the published gold standard, to those treated with FBS. Accordingly, it was not surprising that in a whole transcriptome analysis the three differentiation stages were clearly separable, whereas the different sera clustered strongly together similar to previous results [17]. This is not astonishing, since across a whole population of cells of the same lineage, only minute shifts in gene expression will suffice to induce the changes in protein expression and morphology such as observed by flow cytometry. This emphasises that all four sera delivered equine MoDC of some quality.

It is important to consider that ultimately, DC are not defined by the presence or absence of certain markers, but by their functional ability to stimulate T cells. Here, HS clearly demonstrated an advantage through not inducing non-specific proliferation. This result, observed with all batches of FBS, may reflect their xenogenous and antigenetic nature, with T cells in adult horses reacting against foreign serum components or exogenous agents. To exclude the latter we tested the FBS batches for the presence of pestivirus RNA (a common contaminant of FBS) and can exclude this as a factor. However, as all horses in this study were regularly vaccinated, a sensitisation to foreign proteins present in these vaccines may well have occurred and has been described for equine vaccines before [43]. The strongest proliferation was induced in the heterologous mixed leukocyte reaction (MLR) followed by an antigen-specific recall response against tetanus toxoid. This was to be expected compared to a primary antigen like OVA, where the response relies on the activation of naïve rather than memory T cells. Using the stimulation index to determine the specific reaction above the background, the best performance was observed with HS.

## Conclusion

It can be concluded that eqMoDC generated in the presence of HS showed improved morphological characteristics, higher cell viability and were superior with a more robust performance in the functional T cell assays. While HS did not perform significantly better in all assays, it is the mixture of results that favours HS for the generation of eqMoDC. While PAA’s FBS (A15-101) did not perform worst in all experiments, its inferior performance in morphology and viability assays and the lack of clarity surrounding its composition and thereby functional reproducibility exclude this product completely from use [19]. While FBS can in general be considered further for in vitro research, the results here re-emphasize the need for batch testing. These results are very encouraging for the clinical application of equine MoDC and confirm a most recent report using cells generated with horse serum for recall responses [44]. However, the effect of autologous serum or different serum conditions on the phenotype and function of equine MoDC had not been systematically investigated previously. Prior to clinical application, though horse sera need to be tested extensively for extraneous agents, like the widespread equine hepaciviruses and or treated for inactivation of such. Thus, the serum free generation of MoDC would be desirable, but this has proven inefficient, resulting in very limited cell numbers [8, 45] and is still a matter of debate [24–26, 46, 47]. With the recent progress in defining serum free media for various purposes (discussed in [48]) this goal can be achieved in the near future, but requires further studies to ensure good compliance with MoDC functionality as well.
